# Edmund Comer Board

**Published:** 1924-07

**Authors:** 


### Obituary.

---

### EDMUND COMER BOARD.

We regret to record the death at the advanced age of 85 of Mr. E. C. Board, who was probably the oldest member of the medical profession in Bristol. Born on July 14th, 1838, he was a son of Mr. John Board of Burnham-on-Sea, and received his early education at Harrison's School, Grosvenor, Bath. He became a student at the Bristol Medical School in 1854, and was resident pupil at the Bristol Royal Infirmary. He qualified as M.R.C.S. in 1859 and L.S.A. in 1860, and nine years afterwards, in 1869, he took the L.R.C.P. London. Having served some years as House Surgeon at the Royal Infirmary, he was elected Assistant Surgeon in 1869 and Surgeon in 1871. For twenty-one years he did valuable work in that office, and when he retired in 1892 was appointed Consulting Surgeon. For thirteen years he was the Senior Surgeon, and his old colleagues will bear witness to the diligence and punctuality with which he carried out his duties.

Mr. Board was intensely conservative in carrying out old rules and customs, thus up to the day of his retirement he always went round the wards in his hat. His record is probably unique, for his official association with the Infirmary extended over no less than 55 years.

Mr. Board filled the office of Lecturer on Anatomy in the Old Bristol Medical School in St. Michael's, and lectured there on Forensic Medicine. He was for many years Hon. Sec. to the Bath and Bristol Branch of the British Medical Association, and was subsequently elected President of the Branch. He found a further outlet for his energies as Surgeon to the old Bristol Naval Artillery Volunteers, in the development of which he was closely concerned. For many years he had one of the largest practices in Clifton, and was greatly trusted by a large circle of patients.

A devoted Churchman, Mr. Board acted for some time as Churchwarden of Christ Church, Clifton. His funeral service took place at Emmanuel Church, where a large number of his colleagues and friends were assembled.

